# Supplementary material for: Age at diagnosis, glycemic trajectories, and responses to oral glucose-lowering drugs in type 2 diabetes in Hong Kong: A population-based observational study
Source: PLoS Med. 2020 Sep 18;17(9):e1003316. doi: 10.1371/journal.pmed.1003316 (PMC7500681; doi:10.1371/journal.pmed.1003316)
Supplement: S1 Appendix — (PDF) [file pmed.1003316.s001.pdf]

Appendix 1. Additional Methods

A.

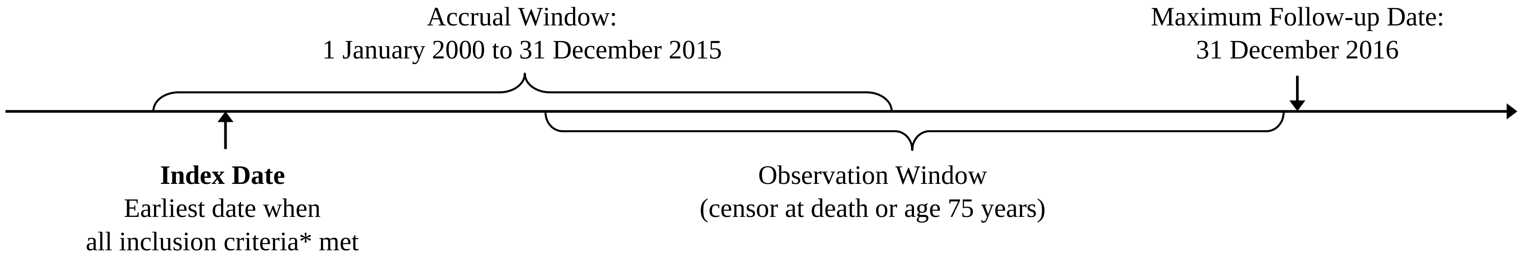

B.

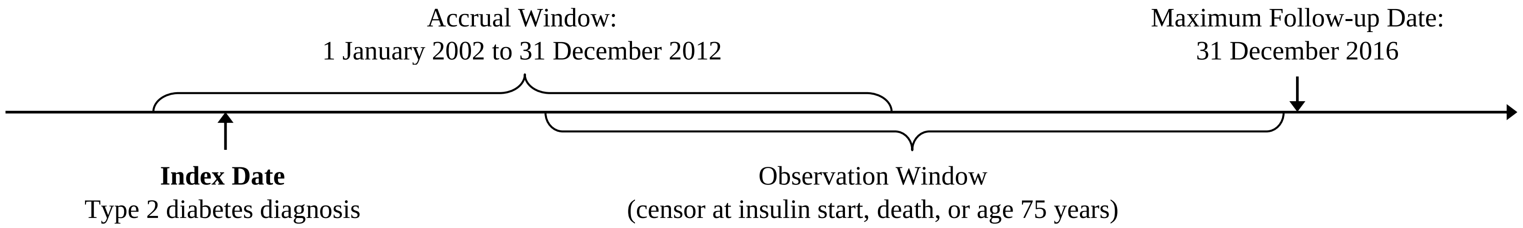

Fig A. Time frame definitions for the Register (A) and Population (B) cohorts.

*Inclusion criteria: age 18 years with type 2 diabetes

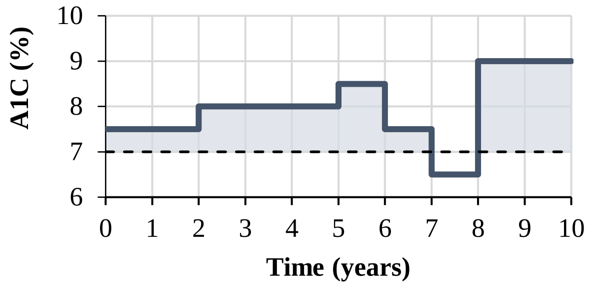

$$
\begin{aligned}
AUC &= 0.5\times2 + 1\times3 + 1.5\times1 + 0.5\times1 + 2\times2 \\
&= 1 + 3 + 1.5 + 0.5 + 4 \\
&= 10 \text{ A1C-years}
\end{aligned}
$$

Fig B. Schematic diagram illustrating the procedure to calculate glycemic exposure, defined as the area under the curve (AUC) of the mean yearly A1C over time, for a hypothetical person with diabetes. The AUC includes only the values in excess of 7%; values <7% are excluded from the calculation. We applied this concept to the Register cohort by plotting the mean A1C in each age at diagnosis group for each year (by attained age) from the mean observed age at diagnosis until age 75 years. The observation period was defined as a constant, calculated as the duration between the mean observed age at diagnosis to age 75 years.

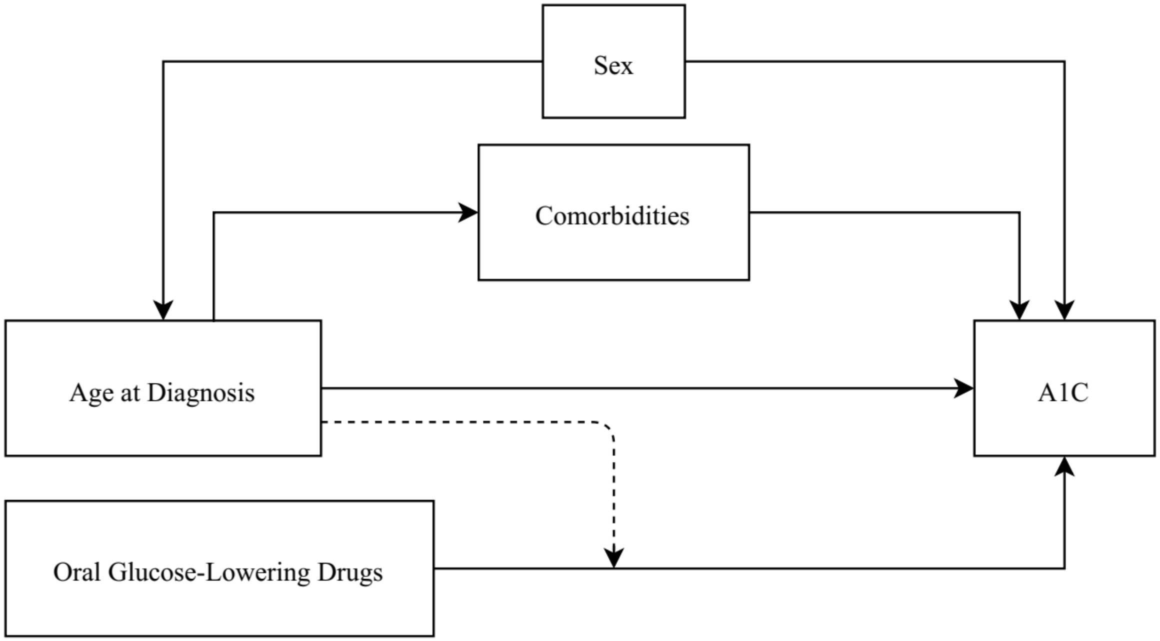

Fig C. Causal diagram depicting the relationship between the age at diagnosis (primary exposure), oral glucose-lowering drugs (secondary exposure), and hemoglobin A_{1c} (A1C; outcome). The causal pathway of interest is the biological relationship between age at diagnosis and A1C. Sex is a confounding variable. Comorbidities (Appendix Table 2) are mediating variables, unrelated to the causal pathway of interest. The hypothesized interaction between age at diagnosis and drug effects is indicated with a dotted line.

Parameterization of Mixed Effects Models

Underlying A1C

We modeled the value of A1C (Y) for each observation i nested within each person j as shown in the basic equation below, where β is the vector of each regression coefficient, and ε is the vector of errors.

$$Y_{ij} = \beta_{0j} + \beta_{1j}time + \varepsilon_{ij}$$

β_{0j} is the intercept for person j (equivalent to A1C when time=0), and β_{1j} is the rate of change of the A1C for each unit of *time*.

For example, consider a hypothetical person j, whose A1C is measured at baseline and annually thereafter. The table below shows the A1C values. In the plot below, each dot represents an A1C measurement, and the blue line connects all dots to display the trend in A1C over time. The blue line's intercept β_{0j} = 6.5 and the slope β_{1j} = 0.5:

$$Y_{ij} = \beta_{0j} + \beta_{1j}time + \varepsilon_{ij}$$

Time (years)	A1C (%)
0	6.5
1	7.0
2	7.5
3	8.0
4	8.5
5	9.0
6	9.5
7	10.0
8	10.5

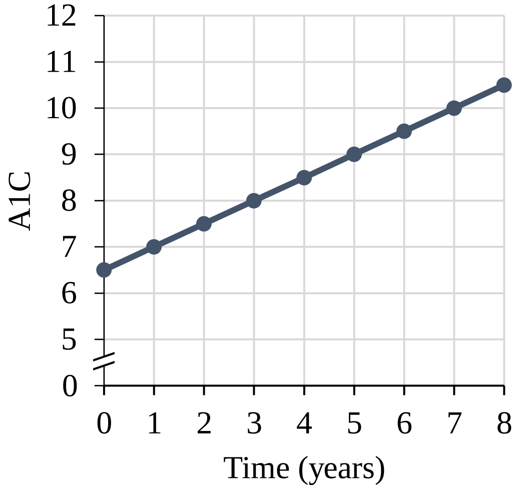

Time-Varying Drug Prescriptions

Next, suppose person j starts taking drug A at $time$ = 4, as shown in the table below. We define the time-varying binary variable *drugA* as equal to 1 when Drug A is active and 0 when Drug A is inactive. In the plot below, the blue dots are A1C measurements taken while Drug A was inactive, and the red dots are those taken while Drug A was active. The blue line connects the blue dots, representing the underlying A1C trend (no drug) and the red line represents the A1C trend on Drug A. As displayed by the downward shift in the red line from the blue line, Drug A drops the A1C by 1% from its expected value without treatment ($\beta_{2j} = -1$):

$$Y_{ij} = \beta_{0j} + \beta_{1j}time + \beta_{2j}drugA + \varepsilon_{ij}$$

Time (years)	A1C (%)	Drug A
0	6.5	0
1	7.0	0
2	7.5	0
3	8.0	0
4	7.5	1
5	8.0	1
6	8.5	1
7	9.0	1
8	9.5	1

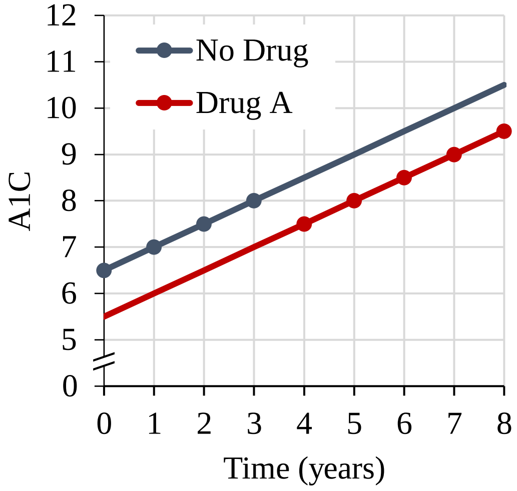

Medication Adherence

The A1C still falls by 1% whenever the drug (prescription) is active, as shown in the plots below:

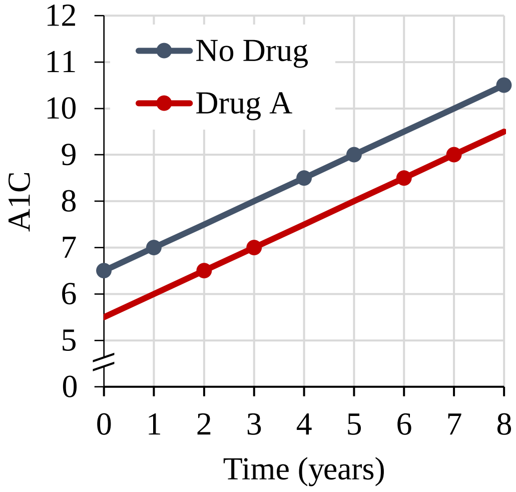

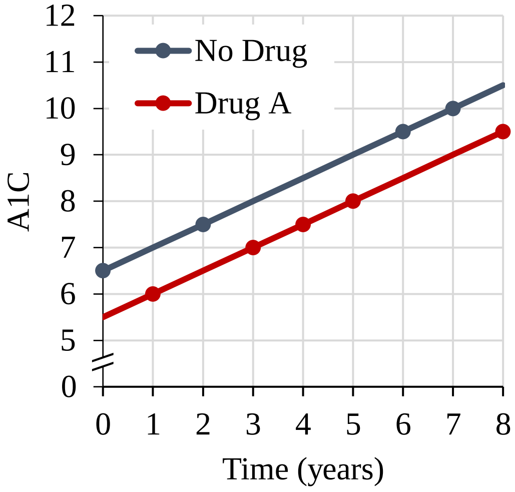

Switching Drugs

Suppose that Drug A is switched to Drug B. We can account for this by defining an additional time-varying binary covariate *drugB*, which indicates when Drug B is active. Based on the table and plot below, Drug B (green) drops the A1C by 1.5% from its expected value without treatment ($\beta_{3j} = -1.5$):

$$Y_{ij} = \beta_{0j} + \beta_{1j}time + \beta_{2j}drugA + \beta_{3j}drugB + \varepsilon_{ij}$$

Time (years)	A1C (%)	Drug A	Drug B
0	6.5	0	0
1	7.0	0	0
2	7.5	0	0
3	8.0	0	0
4	7.5	1	0
5	8.0	1	0
6	8.5	0	1
7	9.0	0	1
8	9.5	0	1

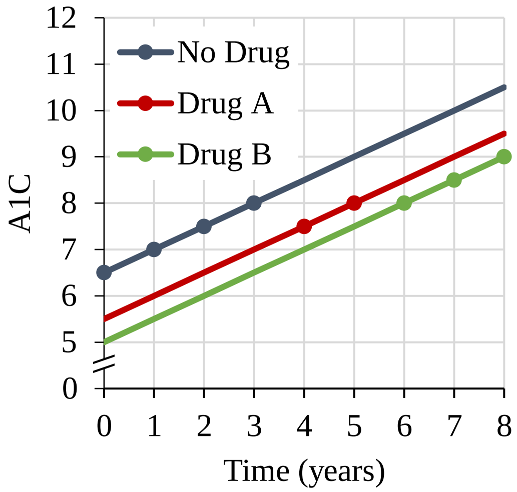

Drug Combinations

Suppose that Drug B is added to Drug A. Clinically, the A1C-lowering effect of a two-drug combination is less than the sum of the A1C-lowering effects of each drug taken independently. For example, drug A may drop the A1C by 1%, and drug B by 1.5%, but the combination of drugs A and B may only drop A1C by 2%. We account for this by defining each drug combination as a unique covariate. In the example below, person *j* starts on Drug A at year 2, switches to Drug B at year 4, then switches to combination A + B (variable *drugAB*, black) at year 6. The combination drops the A1C by 2% ($\beta_{4j} = -2$), while drugs A and B drop A1C by 1% and 1.5% respectively:

$$Y_{ij} = \beta_{0j} + \beta_{1j}time + \beta_{2j}drugA + \beta_{3j}drugB + \beta_{4j}drugAB + \varepsilon_{ij}$$

Time (years)	A1C (%)	Drug		
		A	B	A+B
0	6.5	0	0	0
1	7.0	0	0	0
2	7.5	1	0	0
3	8.0	1	0	0
4	7.5	0	1	0
5	8.0	0	1	0
6	8.5	0	0	1
7	9.0	0	0	1
8	9.5	0	0	1

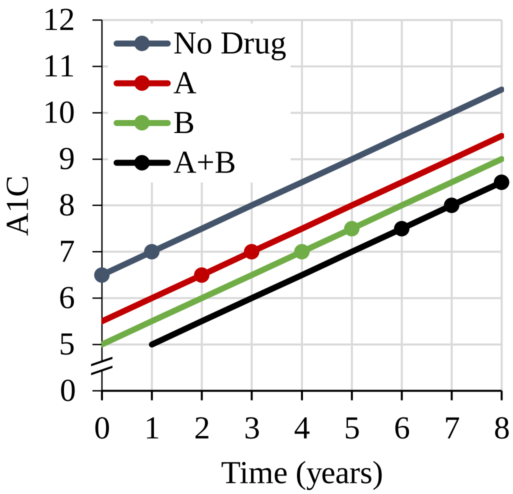

In terms of single drugs, we defined drug terms for metformin and sulfonylureas as these were the most common first-line agents for monotherapy. We excluded other drugs for monotherapy as they were infrequently used (<0.2% of A1C observations). We additionally included the 10 most common metformin- or sulfonylurea-based combinations (2 or 3 drugs) shown in Table A.

Table A. Frequency of non-insulin prescriptions (single drugs and combinations) among A1C observations (including the first observation on each new prescription). The monotherapy and combination therapies selected for the study are indicated with an asterisk (*); all other therapies were categorized as “other”. We only considered combinations metformin or a sulfonylurea for inclusion in the study. Sodium-glucose cotransporter-2 inhibitors were not available in Hong Kong until 2015 [1], so there were insufficient data to evaluate this class of medications.

Drug(s)	Frequency	Percent	Cumulative Percent
M*	976,520	34.61	34.6
S*	417,038	14.78	49.4
Other monotherapy	5,374	0.19	49.6
MS*	1,319,119	46.75	96.3
MSD*	50,487	1.79	98.1
MSA*	12,642	0.45	98.6
MD*	10,855	0.38	99.0
MST*	8,973	0.32	99.3
SD*	7,041	0.25	99.5
SA*	3,799	0.13	99.7
MT*	1,909	0.07	99.7
ST*	1,624	0.06	99.8
MA*	1,134	0.04	100.0
Other combination	5,350	0.18	100.0

Abbreviations: M, metformin; S, sulfonylurea; D, dipeptidyl peptidase-4 inhibitor; A, acarbose; T, thiazolidinedione; G, glucagon-like peptide 1 agonist

Comorbidities

Table B. Comorbidity definitions based on International Classification of Diseases, Ninth Revision (ICD-9) codes listed under the principal diagnosis for hospitalizations occurring within 2 years prior to the index date

Comorbidity	ICD-9 Code(s)
Ischemic Heart Disease	410–414
Congestive Heart Failure	428
Stroke	430–438
Peripheral Arterial Disease	250.7, 443.8, 443.9, 785.4, 895–897
Cancer	140–240

Appendix 2. Additional Results

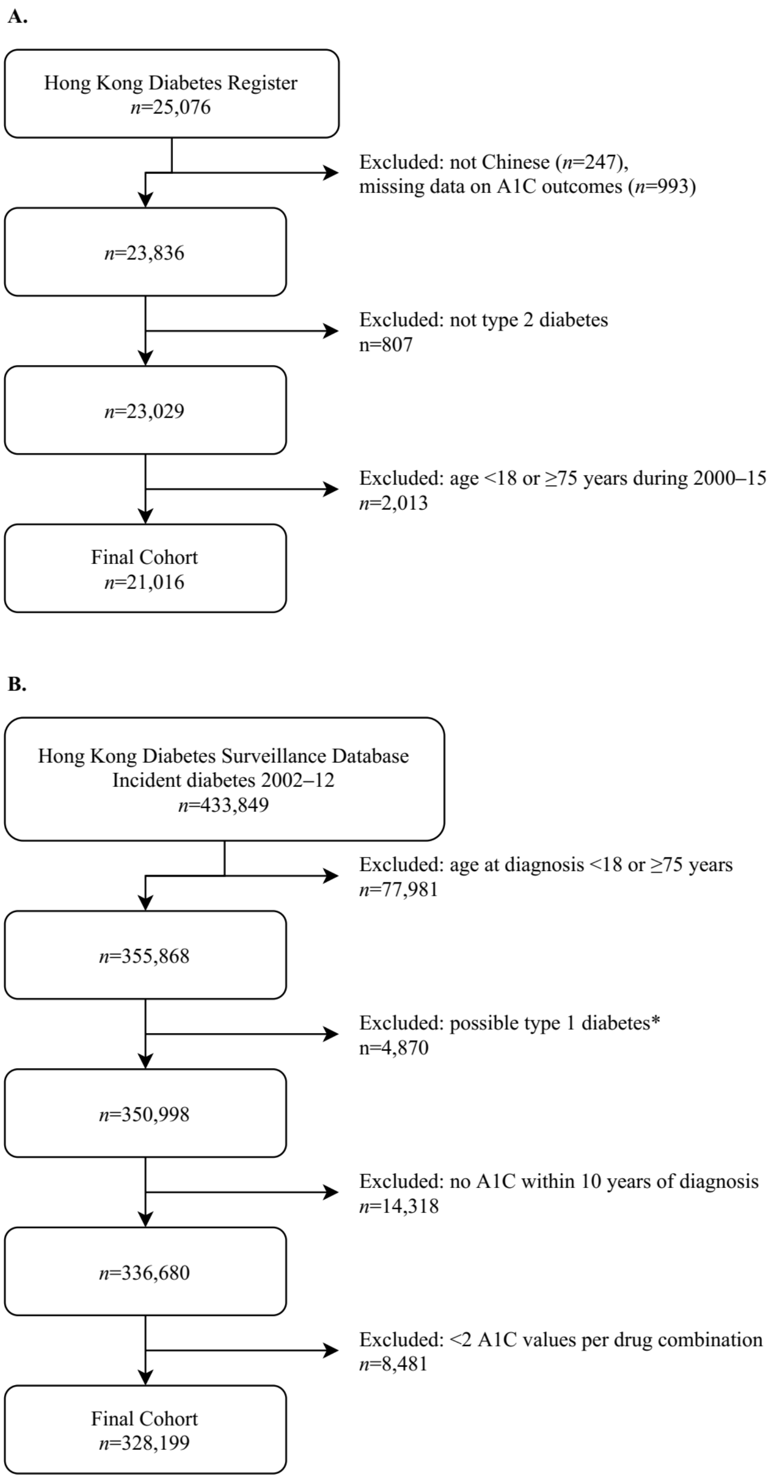

Fig D. Flow diagrams depicting creation of the Register (A) and Population (B) cohorts

*Insulin prescription occurring within 90 days of diagnosis (excludes 93.0% of T1D [2])

Table C. Glycemic deterioration over the first 10 years after type 2 diabetes diagnosis, stratified by age at diagnosis, in the Hong Kong Diabetes Surveillance Database (2002–16). Glycemic deterioration is the modelled slope of the A1C over time after adjusting for oral glucose-lowering drug prescriptions. See Figure 2 for sample sizes.

Age at Diagnosis (years)	Glycemic Deterioration (% per year; 95% confidence interval)
20	+0.0809 (0.0780, 0.0838)
25	+0.0703 (0.0677, 0.0728)
30	+0.0596 (0.0574, 0.0618)
35	+0.0490 (0.0471, 0.0508)
40	+0.0383 (0.0368, 0.0399)
45	+0.0277 (0.0265, 0.0289)
50	+0.0170 (0.0161, 0.0180)
55	+0.0064 (0.0056, 0.0072)
60	−0.0043 (−0.0050, −0.0035)
65	−0.0149 (−0.0158, −0.0140)
70	−0.0256 (−0.0267, −0.0244)

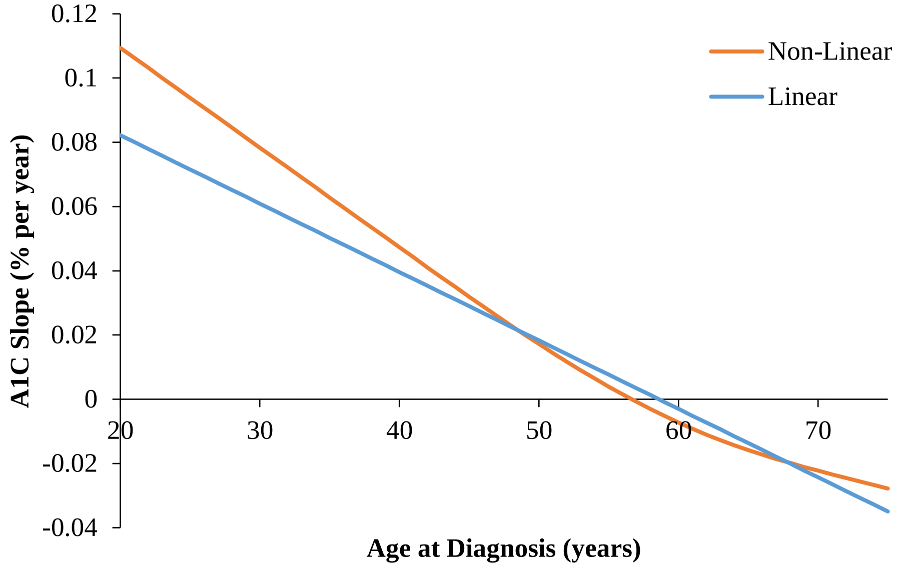

Fig E. Relationship between A1C slope versus age at diagnosis using the linear and non-linear models

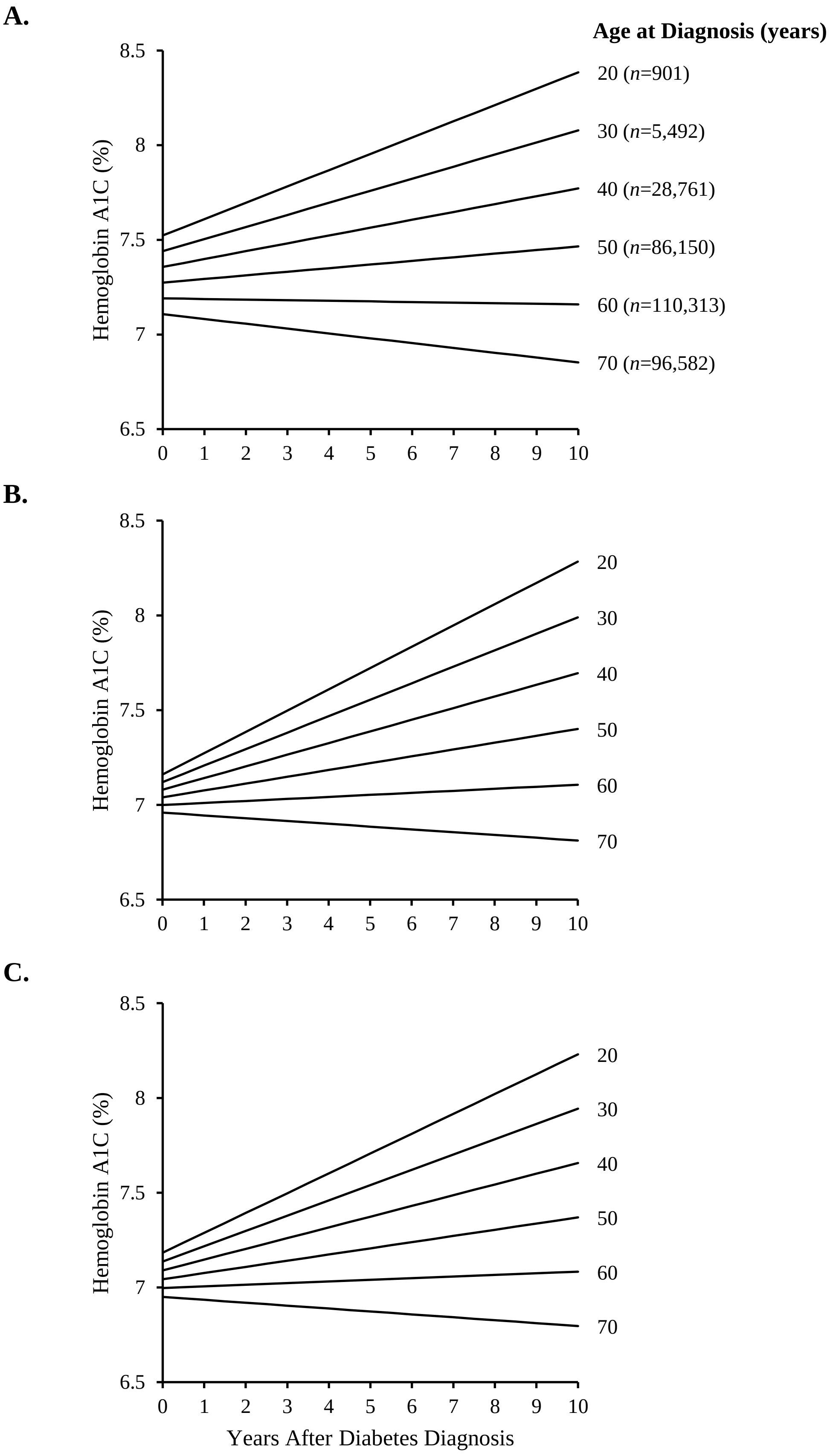
A.
Age at Diagnosis (years)
20 (n=901)
30 (n=5,492)
40 (n=28,761)
50 (n=86,150)
60 (n=110,313)
70 (n=96,582)
Hemoglobin A1C (%)
8.5
8
7.5
7
6.5
0 1 2 3 4 5 6 7 8 9 10
B.
20
30
40
50
60
70
Hemoglobin A1C (%)
8.5
8
7.5
7
6.5
0 1 2 3 4 5 6 7 8 9 10
C.
20
30
40
50
60
70
Hemoglobin A1C (%)
8.5
8
7.5
7
6.5
0 1 2 3 4 5 6 7 8 9 10
Years After Diabetes Diagnosis

Fig F. (previous page) Glycemic deterioration over time among insulin-naïve people with type 2 diabetes, stratified by age at diagnosis (2002–16). Glycemic deterioration is the slope of the A1C over time after adjusting for oral glucose-lowering drug prescriptions.

(A) Original model including all A1C values

(B) Model excluding A1C values from the first 6 months

(C) Model excluding A1C values from the first 12 months

A.

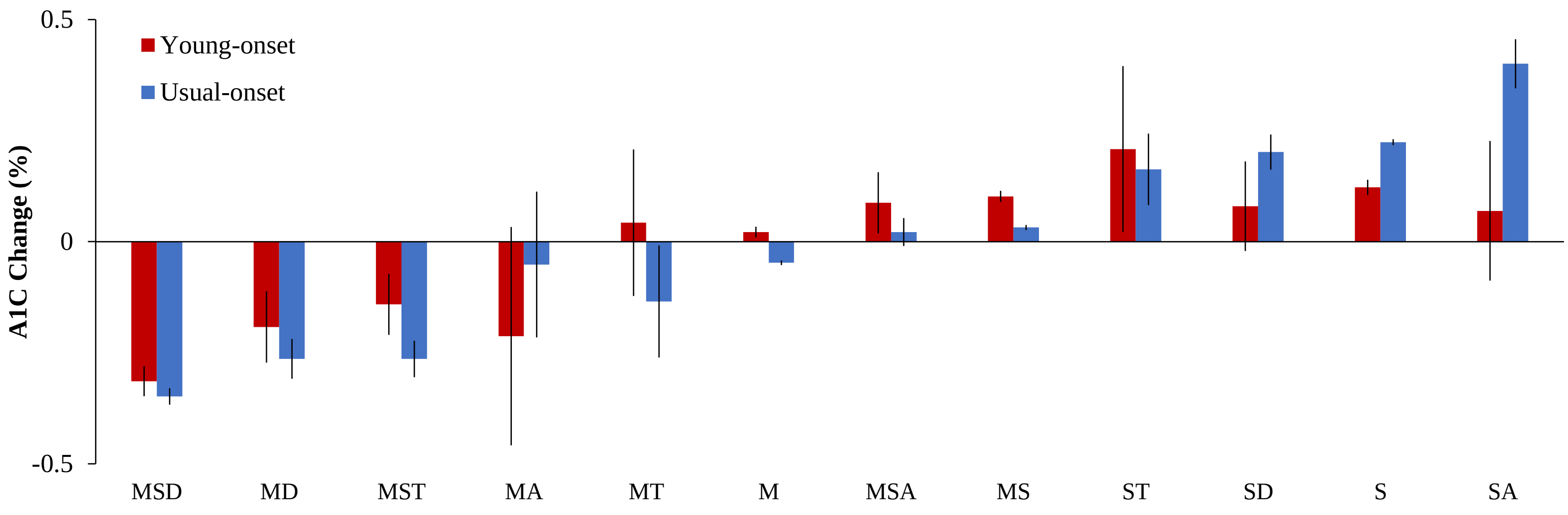
0.5
0
-0.5
A1C Change (%)
Young-onset
Usual-onset
MSD
MD
MST
MA
MT
M
MSA
MS
ST
SD
S
SA

B.

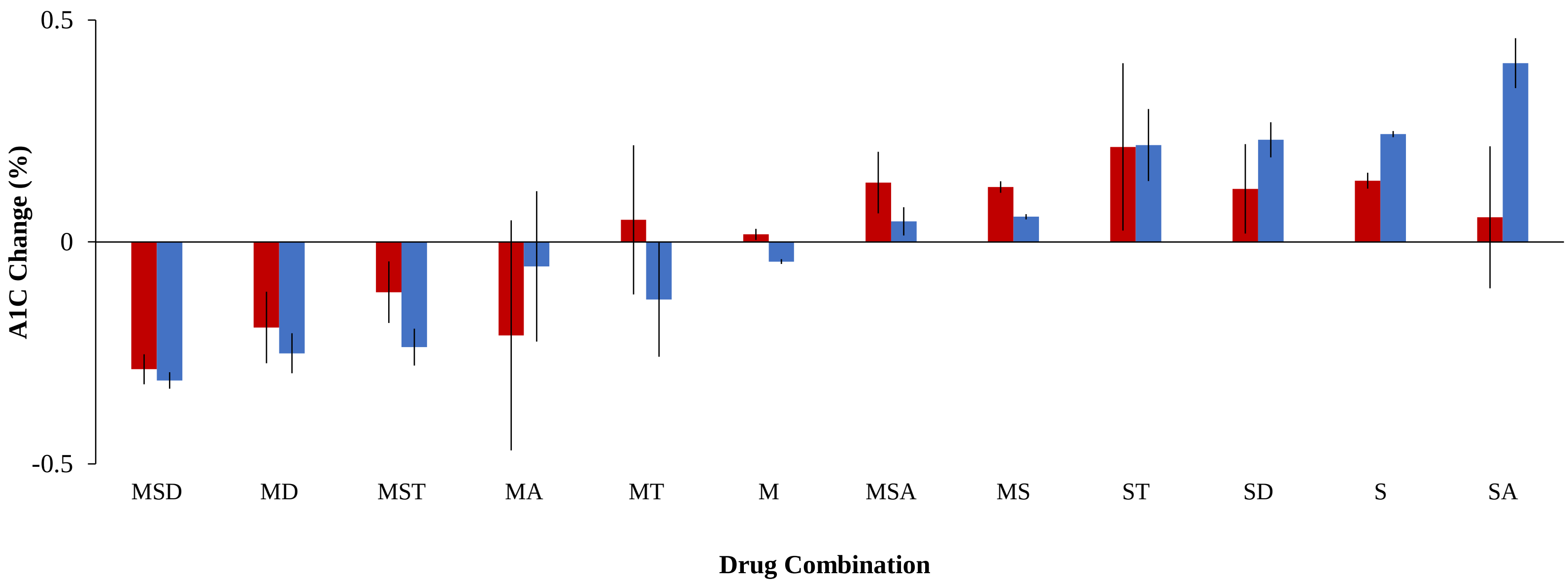
0.5
0
-0.5
A1C Change (%)
MSD
MD
MST
MA
MT
M
MSA
MS
ST
SD
S
SA
Drug Combination

Fig G. (previous page) A1C responses to various oral glucose-lowering drug combinations among insulin-naïve people with young-onset type 2 diabetes (age at diagnosis <40 years) and usual-onset type 2 diabetes (age at diagnosis ≥40 years) during their first decade after diabetes diagnosis, observed during 2002 to 2016. In this sensitivity analysis, A1C values measured during the first 6 (A) or 12 (B) months were excluded. Error bars indicate 95% confidence intervals. Differences between young- and usual-onset type 2 diabetes were statistically significant across all combinations (omnibus test $p<0.0001$).

Abbreviations: M, metformin; S, sulfonylurea; D, dipeptidyl peptidase-4 inhibitor; T, thiazolidinedione; A, acarbose

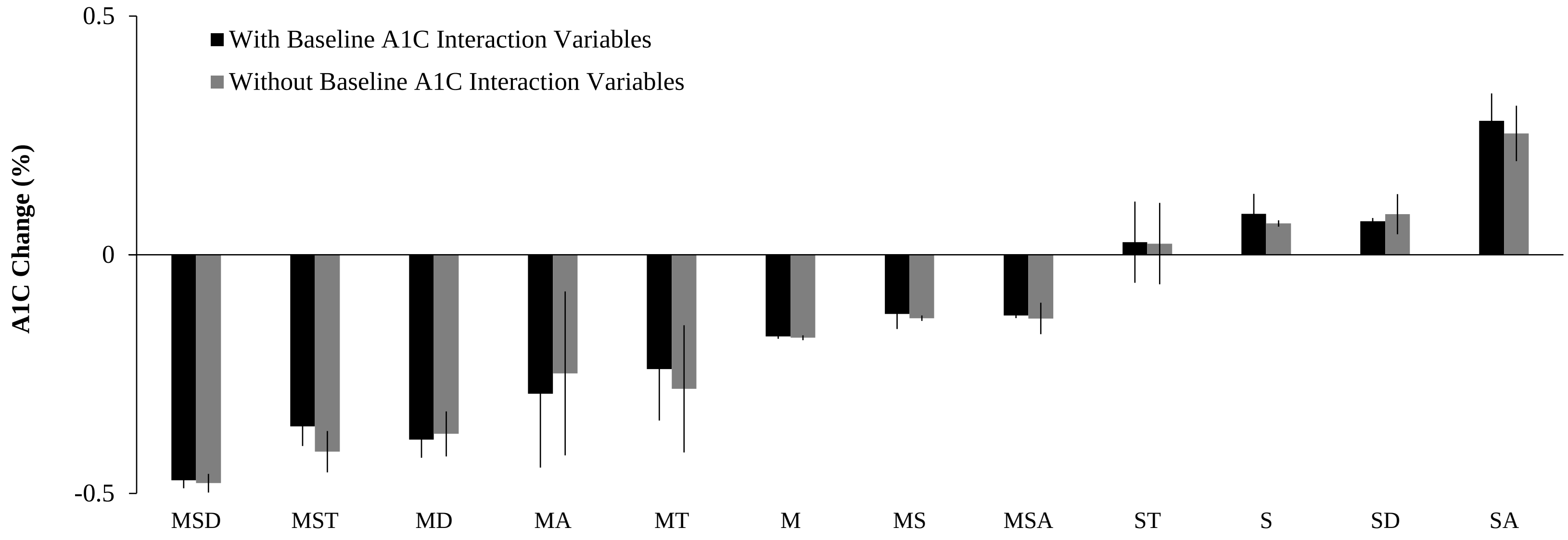

Fig H. A1C responses to various oral glucose-lowering drug combinations during their first decade after diabetes diagnosis among insulin-naïve people with type 2 diabetes, observed during 2002 to 2016. In this sensitivity analysis, we included an interaction term for each drug combination with the baseline A1C for that combination. Results are shown for a baseline A1C level of 7.5%. This model excluded interaction terms with each drug combination and age at diagnosis due to computational limitations. The results from the original model (without baseline A1C) for people with usual-onset type 2 diabetes (age at diagnosis ≥40 years) are shown for comparison.

Abbreviations: M, metformin; S, sulfonylurea; D, dipeptidyl peptidase-4 inhibitor; T, thiazolidinedione; A, acarbose

Appendix References

1. Wu H, Lau ESH, Yang A, Ma RCW, Kong APS, Chow E, et al. Trends in diabetes-related complications in Hong Kong, 2001–2016: a retrospective cohort study. Cardiovascular Diabetology. 2020 May 12;19(1):60.

2. Ke C, Stukel TA, Luk A, Shah BR, Jha P, Lau E, et al. Development and validation of algorithms to classify type 1 and 2 diabetes according to age at diagnosis using electronic health records. BMC Med Res Methodol. 2020 Feb 24;20(35):1–15.
