# Supplementary material for: Age at diagnosis, glycemic trajectories, and responses to oral glucose-lowering drugs in type 2 diabetes in Hong Kong: A population-based observational study
Source: PLoS Med. 2020 Sep 18;17(9):e1003316. doi: 10.1371/journal.pmed.1003316 (PMC7500681; doi:10.1371/journal.pmed.1003316)
Supplement: S1 Analysis plan — (PDF) [file pmed.1003316.s002.pdf]

Project Initiation

**Project Title:**	Glycemic Burden and Treatment Effectiveness in Young-Onset Type 2 Diabetes: A Real-World Study of over 300000 Adults
**Project Objectives:**	To determine the following, in a Chinese adult population:<br>Part 1<br>1. What is the average **achieved** A1C among people with incident and prevalent T2D?<br>a. What is the cumulative excess glycemic burden (area under the curve of achieved A1C over time, with excess defined as anything over 7%, and no adjustments for any medications) over the lifespan (prevalent cohort), and does this vary when stratified by age of onset?<br>Part 2<br>2. What is the underlying rate of A1C change (a surrogate for beta-cell deterioration) among people with incident T2D followed for up to 10 years?<br>a. Does this rate vary by age of onset?<br>3. What is the effectiveness (ATT, average treatment effect among the treated) in A1C lowering of each class/combination of non-insulin antihyperglycemic agents (metformin, SU, acarbose, TZD, DPP4 inhibitors, and all combinations) on A1C?<br>a. For each class/combination, does the ATT vary by age of onset?<br><br>**Terms/Definitions:**<br>young-onset type 2 diabetes (YOD): type 2 diabetes with onset age <40 years<br>usual-onset type 2 diabetes (UOD): type 2 diabetes with onset age >= 40 years<br><br>**Hypotheses:**<br>I hypothesize that beta cell decline and glycemic burden are much higher in YOD compared to UOD, and that non-insulin agents do not reduce A1C more effectively in YOD.
**Principal Investigator (PI):**	Calvin Ke
**Project Team Member(s)**	Calvin Ke, Juliana Chan, Andrea Luk, Eric Lau, Baiju Shah, Thérèse Stukel
**DCP Creation Date and Author:**	***Date*** ***Name***<br>2018 August 23 Calvin Ke

Data	
Datasets	
**Hong Kong Diabetes Surveillance Database** (HKDSD, "**incident cohort**," n≈800000): contains secondarily collected health administrative data purchased from the publicly-administered Hong Kong Hospital Authority (HA). Only individuals with diabetes are included. People followed in private clinics are not captured unless they have used HA services (hospital, laboratory, prescription from an HA physician). Diabetes is defined using laboratory definitions and prescriptions. Diabetes subtype is classified using an algorithm involving prescriptions and hospitalizations (validated in a separate study). Laboratory data, diabetes-related prescriptions, hospitalization records, and date of death are included. This dataset does not include socioeconomic status, addresses, smoking, blood pressure, or body mass index. The HA dataset is provided in an anonymized format, and all dates are only accurate to the month.	2000-2015
**Hong Kong Diabetes Register** (HKDR, "**prevalent cohort**," n≈22000): contains all hospitalizations, demographic, clinical, laboratory data for diabetes patients seen at HA clinics for "comprehensive diabetes assessment" within a sub-cluster of hospitals in Hong Kong (New Territories East Cluster). Essentially, the HKDR contains a subset of people in the HKDSD. Although sample size is smaller, the comprehensive assessments provide additional data on clinically-classified diabetes subtype, education (assessments after 2007), employment, smoking, blood pressure, diet, physical activity, and body mass index. The assessment typically occurs 3-5 years after diabetes onset, and asessments may repeated every 3-5 years.	2000-2015

Project Amendments and Reconciliation			
DCP Amendment History (add additional rows as needed):	***Date***	***Name***	***Amendment***
	19 September 2018	Calvin Ke	• Post-insulin cohort moved to a different project<br>• 12 combinations of drugs selected as most clinically relevant based on project team discussion<br>• Model parameterization finalized based on project team discussion

Project Cohort

**Study Design**	☒ Cohort study ☐ Matched cohort study ☐ Case-control study<br>☐ Cross-sectional study ☐ Other (specify):
**Index Event / Inclusion Criteria**	1. Age 18-75 years<br>2. Diagnosed with type 2 diabetes (different dates for incident/prevalent cohorts)<br>3. Ethnic Chinese resident of Hong Kong (only for prevalent cohort in HKDR, where ethnicity and residency are identified)
**Estimated Size of Cohort (if known)**	

**Exclusions (in order)**	*Step*	Description
	1	Type 1 diabetes: criteria as defined in previous validation study
	2	
	3	

Project Time Frame Definitions – Incident Cohort

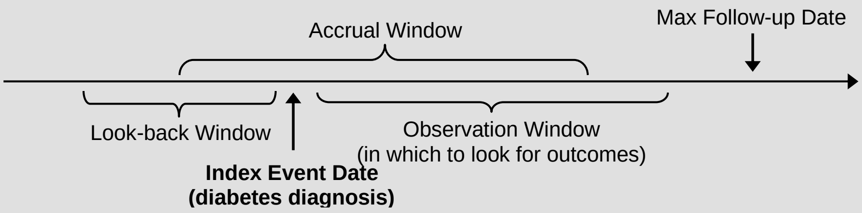

**Accrual Start/End Dates**	January 1, 2002 to December 31, 2012
**Max Follow-up Date**	December 31, 2015
**When does observation window terminate?**	Maximum of 10 years (minimum of 3 years); censor at death
**Lookback Window(s)**	2 years prior to index

Project Time Frame Definitions – Prevalent Cohort

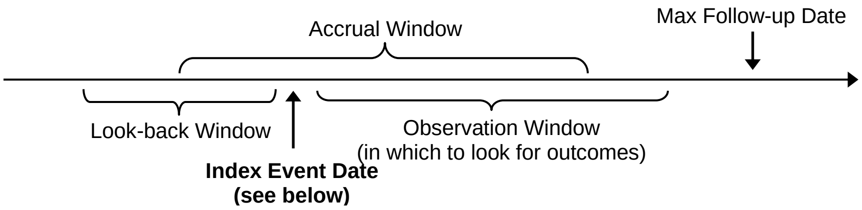

**Index date**	First date on which patient is fulfills all inclusion criteria: age 18, diagnosed with type 2 diabetes, ethnic Chinese resident of Hong Kong
**Accrual Start/End Dates**	January 1, 2000 to December 31, 2015
**Max Follow-up Date**	December 31, 2015
**When does observation window terminate?**	December 31, 2015, censor at death
**Lookback Window(s)**	N/A

Part 1 is a descriptive analysis of the prevalent cohort. It is omitted from the next two sections because there is no modeling involved.

Variable Definitions (PART 2)

**Main Exposure or Risk Factor**	**Onset age**<br>• Expressed as a continuous variable. For clarity of presenting the results, effect sizes for YOD versus UOD are calculated as the effect of a 20-year difference in onset age to match the observed onset age difference between these groups (34 vs. 54 years)
**Secondary Exposures**	Treatment with each non-insulin medication class and combination (classes: metformin, sulfonylureas, acarbose, TZD, DPP-4 inhibitors, GLP-1 agonists).<br>• The A1C lowering effect of each drug typically decreases when used in combination. As the effects are not additive, each unique combination of classes will be included as an additional term.<br>o There are 1956 possible unique combinations of drugs. It is likely that there are <25 combinations that are commonly used in practice. Drugs and combinations that are uncommon will be grouped together as “other medications/combinations” to preserve degrees of freedom.
**Primary Outcome Definition**	A1C (pre-insulin initiation, measured at different time points)

Variable Definitions (PART 2)

	*if insulin is started, individuals are censored at the time it is started. Insulin is not included as a drug effect in this part because unlike the other medications, there is no ceiling effect on A1C lowering as there is no maximum dose. The effect of different doses also varies across individuals. While these effects are predictable based on individual characteristics such as BMI, data on doses are incomplete and BMI is only available in the HKDR sub-cohort.
**Secondary Outcome Definition(s)**	
**Baseline Characteristics**	Sex<br>Comorbidities<br>• defined by primary discharge diagnosis of hospitalizations occurring within the 2-year look-back window, as a categorical variable [yes/no] for each of the following 5 diagnoses (based on data availability): ischemic heart disease, congestive heart failure, stroke, peripheral arterial disease, cancer
**Other Variables**	Time (measured in years from the diagnosis date)

Analysis Plan

Descriptive Tables:

Table 1. Baseline characteristics according to primary/secondary exposure

Figure 1. Mean achieved A1C stratified by age of onset

Figure 2. Real-world A1C lowering for various non-insulin antihyperglycemic agents, according to onset age

Statistical Model(s)

**Type of model**	Linear mixed effects model
**Primary independent variable**	Drug class/combination
**Dependent variable**	A1C (pre-insulin only)
**Covariates**	onset-age<br>sex<br>time<br>ischemic heart disease<br>congestive heart failure<br>stroke<br>peripheral arterial disease<br>cancer<br>onset-age* time

**Sensitivity Analyses**	
**Type of model**	Repeat all models to assess whether A1C varies non-linearly with time<br>• Use a restricted spline to model the effect of time on A1C
**Primary independent variable**	
**Dependent variable**	
**Covariates**	
